# Functional Recovery After Stroke and Its Association with Patient’s Quality of Life and Caregiver Burden

**DOI:** 10.3390/jcm15051975

**Published:** 2026-03-04

**Authors:** Barbara Grabowska-Fudala, Krystyna Jaracz, Karolina Filipska-Blejder, Paweł Kleka, Aleksandra Pawlicka, Robert Ślusarz

**Affiliations:** 1 Department of Neurological Nursing, Faculty of Health Science, Poznań University of Medical Sciences, 60-806 Poznan, Poland; jaracz@ump.edu.pl; 2 Neurological and Neurosurgical Nursing Department, Faculty of Health Science, Collegium Medicum in Bydgoszcz, Nicolaus Copernicus University in Toruń, 85-821 Bydgoszcz, Poland; karolinafilipskakf@gmail.com (K.F.-B.); robert.slusarz@cm.umk.pl (R.Ś.); 3 Faculty of Psychology and Cognitive Sciences, Adam Mickiewicz University, 60-568 Poznan, Poland; pawel.kleka@amu.edu.pl; 4 ITTI, 61-612 Poznan, Poland; aleksandra.pawlicka@itti.com.pl

**Keywords:** stroke, recovery, trajectories, functional status, quality of life

## Abstract

**Background**: The process and dynamics of post-stroke recovery can vary considerably across patient subgroups. However, few studies have explored long-term functional recovery profiles (an essential outcome indicator), particularly in relation to patients’ quality of life, and caregiver burden. This study aimed to identify distinct 12-month trajectories of functional recovery among stroke survivors, and to examine their associations with sociodemographic and clinical factors, as well as patient-reported outcomes. **Methods**: The study involved 225 patients with acute ischemic stroke, assessed at admission (T0), discharge (T1), and at 3 (T2) and 12 months (T3) post-discharge. Informal caregivers participated at T2 (*n* = 126) and T3 (*n* = 118). Functional status was measured using the modified Barthel Index, quality of life using the Stroke-Specific Quality of Life scale, and caregiver burden with the Caregiver Burden Scale. Latent growth mixture modelling (LGMM) was applied to identify recovery trajectories. Associations with sociodemographic and clinical variables, quality of life, and caregiver burden were analysed using ANOVA and χ^2^ tests. **Results**: Three recovery trajectory classes were identified: Class 1 (moderate upward, 20.4%), Class 2 (low-stable, 4.9%), and Class 3 (high-functioning stable, 74.7%). Class 3 patients were younger, less impaired at baseline, and more frequently diagnosed with lacunar strokes. Class 2 comprised older, more impaired individuals and had the lowest proportion of males. Class 1 represented intermediate baseline profiles with gradual functional improvement over time. Patient-reported outcomes differed significantly between classes (*p* < 0.001): Class 3 had the highest quality of life and lowest caregiver burden whilst Class 2 consistently reported the poorest quality of life. **Conclusions**: This study demonstrates significant heterogeneity in post-stroke functional recovery and its associations with clinical, sociodemographic, and patient-reported outcomes. Identifying recovery trajectories may support more personalised stroke care and rehabilitation planning.

## 1. Introduction

Stroke remains a leading cause of mortality and one of the main contributors to the global disease burden, as expressed by disability-adjusted life years index (DALYs) [1]. Its consequences extend significantly beyond the early phase of the neurological event, having long-term effects not only on patients’ functional status but also on their quality of life and the well-being of their families due to the substantial burden of caregiving [2,3,4]. The ultimate extent of these impacts is largely determined by the course of recovery [5,6]. Recovery after stroke is a complex and multidimensional process that requires coordinated rehabilitation and comprehensive support both during the period of early neuroplastic changes and in longer-term adaptation phases [7]. Effective rehabilitation therefore aims not only to restore functional abilities but also to enhance patients’ overall quality of life and minimise the burden on family caregivers [8].

Functional recovery after stroke, often operationally defined as improvement in activities of daily living [9], is known to be heterogeneous [10]. It may be influenced by multiple factors, such as stroke severity, stroke subtypes, age, comorbidities, and individual adaptation capacities [9,10,11]. However, traditional group-based analyses tend to report mean-level outcomes [12], assuming homogeneous recovery pathways across patients, which may overlook clinically meaningful individual differences [13].

To better capture this heterogeneity, person-centred statistical approaches such as latent class analysis and latent class growth modelling have gained increasing attention. These methods enable the identification of distinct subgroups of patients with similar recovery patterns over time [14], offering a more nuanced understanding of recovery. This knowledge can be essential for optimising rehabilitation strategies, allocating resources effectively, and tailoring support for both patients and their families.

Although trajectory-based approaches have been used to explore recovery patterns in domains such as motor function, physical activity, depression, and quality of life [10,15,16,17,18], their application to functional recovery in terms of ADLs from the acute phase onward remains limited [19,20]. Moreover, previous studies have rarely examined how distinct ADL recovery trajectories translate into differences in patient quality of life and caregiver burden over time. In particular, existing trajectory research has either focused on determinants of functional classes [19], or on hard clinical endpoints [17,20], without integrating downstream patient and caregiver-reported outcomes into the same longitudinal framework. Importantly, although some research has modelled trajectories of post-stroke quality of life and examined their associations with caregiver outcomes, these analyses have focused on QoL trajectories per se, beginning at the rehabilitation discharge stage, rather than on functional recovery trajectories from stroke onset and their subsequent psychosocial consequences [18]. Thus, the directional link between early functional recovery patterns and later patient and caregiver outcomes remains insufficiently explored.

This represents an important knowledge gap, especially considering that functional independence is one of the key long-term outcome indicators after stroke. Furthermore, while patient-reported outcomes are increasingly recognised as essential in stroke care and rehabilitation [3,21], to the best of the authors knowledge, integrating them with longitudinal patterns of functional recovery remains limited.

Therefore, the present study aimed to (1) identify distinct trajectories of functional recovery among stroke survivors over a 12-month period; (2) examine how these functional recovery trajectories are associated with patients’ sociodemographic and clinical factors; and (3) investigate the relationships between these trajectories and person-reported outcomes, including patients’ quality of life and family caregiver burden.

By linking early functional recovery patterns with subsequent patient and caregiver-reported outcomes within a unified longitudinal design, this study extends previous trajectory research and offers clinically relevant insights for the development of individualised rehabilitation programmes. It further underscores the importance of integrating functional, psychosocial, patient-, and family-related outcomes in a care pathway-oriented approach to post-stroke care planning, as recommended in the Action Plan for Stroke in Europe 2018–2030 [22].

## 2. Materials and Methods

### 2.1. Study Participants

The study was conducted among patients with acute ischemic stroke who were treated at the Department of Neurology and Cerebrovascular Diseases with the Stroke Unit at Dr. Ludwik Bierkowski Clinical Hospital of the Ministry of Internal Affairs and Administration in Poznań between May 2012 and March 2016. Data were collected at four time points. The first assessment (T0) was performed upon hospital admission and focused on the evaluation of the patient’s neurological and functional status. The second assessment (T1) took place at the end of hospitalization and included repeated evaluation of neurological and functional status. The third (T2) and fourth (T3) assessments were carried out in patients’ homes at 3 and 12 months after discharge, respectively. In addition to neurological and functional assessments, the final two follow-ups included evaluations of patients’ quality of life and caregiver burden.

Patients with a confirmed diagnosis of acute ischemic stroke were consecutively recruited into the study. Inclusion criteria were confirmation of ischemic stroke by CT or MRI; absence of comorbid or chronic medical conditions likely to impair functional or cognitive capacity significantly; provision of informed consent; and permanent residence in the Greater Poland region (Wielkopolskie Voivodeship, with Poznań as its capital), which was required for logistical reasons related to the follow-up visits at patients’ homes.

A total of 355 patients were initially enrolled. However, 130 were excluded from follow-up due to severe cognitive or speech impairments, referral to long-term care or another hospital department, death, loss of contact following discharge, or inability to determine their current residence. Ultimately, 225 patients completed all four assessments.

Informal caregivers were also recruited for a subset of the study sample to assess caregiver burden. This evaluation was conducted only with individuals who were the patient’s primary caregivers, were available during the home visit, and provided informed consent. As a result, the caregiver subgroup comprised 126 participants at T2, and 118 participants at T3.

The study was approved by the Bioethics Committee at the Poznań University of Medical Sciences (approval no. 283/12 and date of approval: 1 March 2012) and was conducted in accordance with the principles of the Declaration of Helsinki. All participants provided informed consent during their hospitalization, either in written or oral form, which was documented by the researcher. Participation in the study was voluntary, and participants were informed that they could withdraw at any stage without providing a reason.

### 2.2. Methods

The National Institutes of Health Stroke Scale (NIHSS) was applied to assess the neurological status of patients [23]. The scale is a widely used, standardized tool that involves several domains, including level of consciousness, orientation, following commands, eye movements, visual fields, facial nerve function, motor strength of the upper and lower limbs, ataxia, sensory function, aphasia, dysarthria, and neglect. The total score ranges from 0 to 42 points, with higher scores indicating greater neurological impairment. The scale has demonstrated good inter-rater reliability with Kappa coefficients ranging from 0.66 to 0.77 [24].

The 20-point Barthel Index was applied to assess patients’ functional status. This scale measures performance in 10 activities of daily living, with total scores ranging from 0 to 20 points, where higher scores indicate better functional independence. The tool distinguishes five levels of functional disability: very severe disability (0–4 points), severe (5–9 points), moderate (10–14 points), mild (15–19 points), and no disability (20 points) [25]. This scale has shown satisfactory psychometric properties, including Kendall’s coefficient of concordance of 0.93 across four rating methods [26].

The Stroke-Specific Quality of Life (SS-QOL) scale was used to assess the patients’ quality of life. It consists of 49 items grouped into 12 subscales, covering two domains: physical (self-care, vision, speech, mobility, work, upper limb function) and psychosocial (thinking, personality, mood, family roles, social roles, energy). The overall score is calculated as the mean of subscale scores and ranges from 1.0 to 5.0, with higher scores indicating better quality of life. The scale demonstrated good internal consistency, with Cronbach’s alpha coefficients ≥ 0.73 for the subscales and the total scale [27,28].

The Caregiver Burden Scale (CBS) was used to evaluate caregiver burden. The CBS consists of 22 items grouped into five dimensions: general strain, isolation, disappointment, emotional involvement, and environment. Responses are rated on a 4-point Likert scale, with higher scores indicating greater burden. Only the overall score was used for analysis. The CBS has demonstrated good internal consistency and construct validity [29,30].

Sociodemographic and clinical data were obtained directly from patients and/or their family caregivers, and from hospital medical records and documented in a study-specific questionnaire.

### 2.3. Statistical Analysis

Descriptive statistics (means, SDs, frequencies, percentages) were calculated to summarize baseline data. To identify distinct functional recovery trajectories across four time points (baseline, discharge, 3-month, and 12-month follow-up), latent growth mixture modeling (LGMM) was applied using the lcmm package in R. This semiparametric approach simultaneously estimates heterogeneous growth trajectories while accounting for within- and between-subject variation.

LGMM was specified with the Barthel Index (BI) as the outcome, modeled as a linear function of time, with random intercept and random slope structures (random = ~Time) and class-specific heterogeneity in both intercept and slope (mixture = ~Time). Model estimation used robust maximum likelihood with empirically adjusted standard errors. The random effects variance–covariance matrix (intercept variance, slope variance, and their covariance) was estimated within the model. To avoid local maxima, the gridsearch procedure was employed with 50 random starts and 30 iterations per start.

The optimal number of latent classes (k = 1–4) was determined using: (1) BIC—lower values indicate better fit-parsimony balance; (2) Entropy (calculated as 1−(mean Shannon entropy/log K), where K is the number of classes)—values ≥ 0.80 indicate adequate class separation; and (3) Lo-Mendell-Rubin LRT (LMR-LRT)—a significant *p*-value (*p* < 0.05) indicates that a k-class model significantly improves fit over a k − 1 class model [31,32]. Following model selection, individuals were assigned to their most likely class based on maximum posterior probability. The overall distribution of posterior probabilities was assessed for classification stability and clarity.

Associations between trajectory classes and sociodemographic (age, gender, relationship status, place of residence) and clinical variables (comorbidities, thrombolysis, stroke subtype, NIHSS at baseline, quality of life, and caregiver burden) were examined using one-way ANOVA for continuous variables and χ^2^ tests for categorical variables. For ANOVA, Tukey’s post hoc test with Holm–Bonferroni correction was applied for pairwise comparisons. For categorical variables, pairwise comparisons were conducted using Fisher’s exact tests with Holm correction.

All tests were two-tailed (α = 0.05). Analyses were conducted in R (version 4.1) using lcmm, lme4, and emmeans packages, and Tibco Statistica (version 13) [33,34].

## 3. Results

### 3.1. Participant Characteristics

The study group comprised 225 patients, their mean age being 66.79 years (SD = 12.53). The majority were male (*n* = 134, 59.6%) and in a relationship (married or in a stable partnership) (*n* = 170, 75.6%). More than two-thirds (*n* = 154, 68.4%) resided in urban areas. Most participants (*n* = 207, 92%) had at least one comorbid condition. According to the Oxfordshire Community Stroke Project (OCSP) classification, the most prevalent stroke subtype was partial anterior circulation infarct (PACI; *n* = 90, 40%), while the least common was posterior circulation infarct (POCI; *n* = 31, 13.8%). The mean NIHSS score at admission was 7.56 (SD = 5.21). Thrombolytic therapy with recombinant tissue plasminogen activator (rt-PA) was administered to 50.7% (*n* = 114) of patients.

Regarding caregivers, the sample at T2 included 21 men (16.67%) and 105 women (83.33%), with a mean age of 57.92 years (SD = 12.00). At T3, the caregiver sample comprised 18 men (15.25%) and 100 women (84.75%), with a mean age of 57.26 years (SD = 11.59). Further results are presented in accordance with the three predefined study aims.

### 3.2. Aim 1: Identification of Functional Recovery Trajectories

As a result of the analysis, three distinct classes of functional recovery were identified based on the Barthel Index (BI) assessed at four time points. Model fit indices indicated that the three-class solution provided the best representation of the data. The Lo-Mendell–Rubin adjusted likelihood ratio test (LMR-LRT) was statistically significant when comparing the two-class and three-class models, suggesting that the three-class model offered a significantly better fit (Table 1). Adding a fourth class did not improve model fit, further supporting the adequacy of the three-class solution.

**Figure 1:**
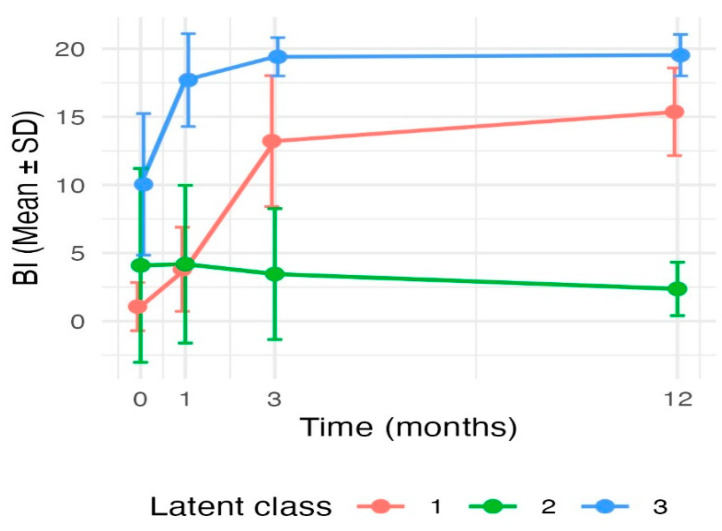
Trajectories of functional recovery as a function of time.

Based on this three-class solution, patients were categorised into three distinct trajectory classes of functional recovery (Table 2 and Figure 1): (1) moderate upward trajectory (red line, *n* = 46, 20.4%): patients in this group started with low baseline functional status, showed rapid improvement during the early post-stroke phase (between baseline and 1 month post-stroke), and continued to improve gradually, reaching a level of functional performance corresponding to mild disability, at the lower end of the BI range at 12 months; (2) low-stable trajectory (green line, *n* = 11, 4.9%): patients in this class demonstrated very limited or no functional independence at baseline with minimal early improvement, and sustained severe functional dependence throughout the 12-month period; (3) high-functioning stable trajectory (blue line, *n* = 168, 74.7%): patients in this group started with relatively high baseline functional status with a rapid, substantial improvement by 1 month, followed by a plateau, maintaining consistently high levels of functional independence up to 12 months post-stroke (Figure 1). Significant differences were observed between the three trajectory classes at all four time points (*p* < 0.001).

### 3.3. Aim 2: Associations Between Recovery Trajectories and Sociodemographic and Clinical Factors

As shown in Table 3, among eight sociodemographic and clinical factors, four were significantly associated with the three recovery trajectory classes: patients’ age, sex, stroke subtype (OCSP classification), and baseline neurological status (NIHSS). Patients in the low-stable class (Class 2) were significantly older than those in the high-functioning stable (Class 3) trajectory (*p* = 0.001). The proportion of male participants was lowest in Class 2 (18.2%) compared with Classes 1 (58.7%) and 3 (62.5%) (*p* = 0.015). Baseline neurological status, measured by the NIHSS, varied significantly across groups (*p* < 0.001), with higher scores observed in Classes 1 and 2 compared with Class 3 (*p* < 0.05). Stroke subtype also differed significantly across trajectory classes, with total anterior circulation infarcts (TACI) being more prevalent in Class 2 than in Classes 1 and 3 (*p* < 0.001). Conversely, the lacunar infarcts (LACI) were significantly more common in Class 3 (37.5%) compared with Classes 1 and 2 (*p* = 0.006). No significant differences were observed in the distribution of partial anterior circulation infarct (PACI) or posterior circulation infarct (POCI) across classes.

### 3.4. Aim 3: Associations Between Recovery Trajectories and Person-Reported Outcomes Including Patients’ Quality of Life and Family Caregiver Burden

As presented in Table 4, quality of life, assessed by SS-QOL scores at both 3 months (T2) and 12 months (T3), differed significantly between the recovery trajectories (*p* < 0.001). At both time points, Class 3 reported the highest scores across all domains, while Class 2 consistently reported the lowest. Class 1 had intermediate scores. Post hoc comparisons showed that Class 3 differed significantly from both Class 1 and Class 2 in all domains at T2 and T3, while Class 1 and Class 2 differed significantly only in the physical and total scores but not in the psychosocial domain. A similar pattern emerged for caregiver burden: at both T2 and T3, caregivers of Class 1 and Class 2 patients reported significantly higher burden scores than those of Class 3 patients. No significant difference in caregiver burden was found between Class 1 and Class 2.

## 4. Discussion

This study identified three distinct groups of patients with different functional recovery trajectories. Two trajectories showed substantial improvement within the first year after stroke, while one trajectory showed persistently low levels of functional status. Across the improving trajectories, the most pronounced gains occurred within the first three months after stroke onset; however, patients in the moderate upward trajectory continued to improve beyond this early period, whereas those in the low-stable trajectory demonstrated no meaningful functional improvement over time. These findings align with the above-mentioned studies (see Introduction) and further confirm the heterogeneous nature of functional recovery following stroke. Du et al. [20] identified four disability patterns within the first year, ranging from no significant disability to persistent severe disability. Similarly, Huang et al. [19] described five trajectory clusters, including a subgroup that remained dependent at 12 months. Comparable results were also reported by Ganesh et al. [35] who also indicated that functional status at three months, as measured by the modified Rankin Scale (mRS), was a predictor of the one-year functional profile, with limited recovery observed beyond this period. Taken together, these findings highlight the need for early identification of patients at risk of a low-stable course to optimise planning of post-acute rehabilitation.

In the present study, most patients were independent or near-independent in activities of daily living, while approximately one quarter were partly or fully dependent. These proportions are broadly comparable to those reported by Huang et al. [19], who found that around 82% of patients regained functional independence, with the remaining 18% dependent at one year post-stroke based on the Barthel Index. Similar proportions of persistent functional dependence measured with the Barthel Index have also been reported in other longitudinal studies [36]. In contrast, studies using the mRS tend to report higher percentages of residual disability; for example, Du et al. [20] identified that around 60% of patients were classified as trajectories with no significant or only slight disability (15.9% and 44.6%, respectively), while approximately 40% followed trajectories indicating moderate to severe disability, of whom 8.7% had persistent severe disability. These discrepancies may partly reflect differences between the outcome measures: the BI focuses on specific basic activities of daily living, whereas the mRS captures broader aspects of functional disability. Thus, while recovery appears favourable overall for many stroke survivors, a substantial proportion of patients continue to experience functional limitations one year after stroke, underscoring the ongoing need for support in daily functioning, including assistance from family caregivers.

Patients in the three trajectory classes differed significantly in several sociodemographic and clinical characteristics. Regarding sex distribution, the two more favourable recovery trajectories showed a similar pattern, with a slightly higher proportion of male patients, whereas the low-stable group included significantly fewer men. This finding corroborates previous evidence suggesting that men are more often represented in favourable one-year recovery trajectories, while women are more frequently found in trajectories characterised by persistent disability [19,20]. This observation is further supported by studies showing that male stroke survivors more often achieve ADL independence than females at 3–12 months and in the longer term post-stroke [37,38,39]. These sex differences may reflect the influence of previously documented factors such as women’s older age at stroke onset, differences in stroke characteristics [40], greater burden of age-related comorbidities, higher likelihood of living alone and being widowed, along with consequently greater social isolation and reduced external support [37,41,42,43].

In addition to sex, age also differentiated between the trajectory classes, with the least favourable course being observed among older patients, which is consistent with previous trajectory-based studies [19,20]. Earlier research suggests that older patients are more likely to show limited functional gains, which may be related to increased comorbidity, reduced neuroplasticity, greater frailty, and shorter periods of early post-stroke improvement [44,45,46].

A borderline association was also found for marital status, with fewer married or partnered individuals in the low-stable trajectory. This finding is in line with previous research indicating that stroke survivors who have a spouse/partner tend to achieve better functional outcomes [47], likely due to ongoing emotional and practical support and more stable underlying resources [48,49]. It should be noted, however, that the stroke literature also emphasizes the importance of the quality of marital relationships [50]. Overall, these findings suggest that the above-mentioned sociodemographic factors such as sex, age, and marital status are interconnected and, when occurring together, may contribute to a less favourable recovery course; nevertheless, this would require confirmation in future studies.

Another factor differentiating the identified recovery trajectories was neurological status at admission. This finding is in agreement with previous evidence indicating that NIHSS score at admission is a strong predictor of post-stroke outcomes [51,52]. By applying a group-based trajectory approach, the present study extends these observations to longitudinal patterns of functional recovery, as assessed by the Barthel Index over the one-year follow-up period.

Finally, differences between the identified recovery trajectories were also observed with respect to the clinical syndrome of stroke. As expected, patients with total anterior circulation infarcts were more likely to follow the least favourable recovery trajectory, which is consistent with previous trajectory-based analyses [19] and outcome studies using OCSP classification [53,54]. Earlier research has shown that TACI is usually associated with larger lesion size [55] which translates into poorer functional outcomes [53], whereas lacunar infarcts are usually linked to more favourable recovery patterns [19,56] particularly in the short and mid-term post-stroke period [57].

With regard to the factors discussed above, the observed associations are based on bivariate analyses and therefore do not account for potential confounding or interrelationships between sociodemographic and clinical variables. Factors such as age, sex, marital status, neurological severity, and stroke subtype may be interdependent, and the identified associations should not be interpreted as independent effects [58,59].

With respect to persons -reported outcomes, the study findings showed that the identified functional recovery trajectories were mirrored in patients’ perceived quality of life, with more favourable functional courses associated with higher quality of life scores. Notably, psychosocial quality of life remained similarly impaired in the moderate upward and low-stable trajectories despite differences in physical functioning, suggesting that psychosocial recovery may progress more slowly than physical improvement after stroke [60]. These findings are congruent with previous research, while approaching the issue from a different analytical perspective. Prior longitudinal studies have primarily focused on identifying trajectories of quality of life and their predictors, demonstrating that functional status was among the significant determinants differentiating favourable and unfavourable QoL trajectories [18,61,62,63]. Overall, results from the present study and existing literature indicate that functional recovery and quality of life constitute interrelated longitudinal processes rather than independent outcomes.

In a similar way, the identified functional recovery trajectories were also reflected in caregiver burden within the subsample of participating caregivers, indicating that less favourable functional courses tended to be associated with higher levels of burden among family caregivers at both 3- and 12-month follow-ups. Although caregiver burden was assessed in a subsample, this pattern is consistent with longitudinal evidence showing that caregiver burden closely follows the stroke survivor’s level of functional dependence and that unfavourable patient trajectories are associated with sustained caregiver strain [62,64]. Furthermore, these findings align with earlier long-term observations indicating that higher patient dependence in activities of daily living may translate into caregiver burden that persists far beyond the first year after stroke [3].

There are several limitations of this study that should be acknowledged. First, the study sample was recruited several years ago, which may limit the generalisability of the findings to current clinical practice. Since then, acute stroke care organisation and treatment, as well as rehabilitation procedures, have progressed substantially, which may influence both early functional trajectories and long-term patient and caregiver outcomes. However, the analyses focused on longitudinal functional recovery trajectories and their associations with sociodemographic and clinical factors and with patient- and caregiver-reported outcomes rather than on the effects of specific therapeutic interventions. Consequently, the identified associations are likely to reflect underlying recovery patterns that remain clinically relevant. Nevertheless, future studies based on more recent cohorts are warranted.

Second, a substantial proportion of initially enrolled patients did not complete all follow-up assessments, partially due to severe cognitive or communication impairments, referral to long-term care, or death. In addition, caregiver burden was assessed only in a subset of patients for whom a primary informal caregiver was available. These factors may have introduced selection bias, with the most severely affected patients and the most burdened caregivers potentially being underrepresented in the final sample. Third, due to the relatively small size of one of the trajectory groups, multivariable analyses aimed at identifying independent determinants of trajectory classes were not performed. As a result, the reported associations between recovery trajectories and sociodemographic or clinical factors are based on bivariate analyses only and may therefore be influenced by potential confounding or interrelationships between predictors. Additionally, the small proportion of participants in this trajectory class may limit the stability and precision of class-specific estimates. Although model fit indices supported the inclusion of this subgroup, and classification quality remained high (mean posterior probability = 0.939; 90.9% of members exceeding the 0.80 threshold), the findings related to this class should be interpreted with caution and require confirmation in larger cohorts.

## 5. Conclusions

In line with research objectives, this study identified three meaningful and distinct trajectories of functional recovery during the first year after stroke: a high-functioning stable trajectory, a moderate upward trajectory, and a low-stable trajectory. In most patients, the pattern of recovery was favourable, with the greatest improvement in the early post-stroke period; however, a smaller but clinically notable subgroup demonstrated persistent dependence in activities of daily living. Older age, female sex, being unmarried, greater neurological impairment at admission, and total anterior circulation infarction were associated with an unfavourable recovery trajectory. Functional recovery was closely aligned with quality of life; however, psychosocial quality of life remained similarly impaired in the moderate-upward and low-stable groups. Identifying individuals at risk of a persistently unfavourable recovery course may facilitate more tailored post-acute care, including prolonged rehabilitation, targeted psychosocial support, and proactive involvement of family caregivers.

## Figures and Tables

**Table 1:** Model fit indices for latent trajectory classes (*n* = 225).

Model	*n*	Percent (%)	BIC	Entropy	LMR-LRT	*p*-Value
**1 class**	225	100	5749.359			
**2 classes**	54	24	5684.344	0.90	81.26	<0.001
	171	76				
**3 classes**	46	20.4	5668.970	0.91	31.62	<0.001
	11	4.9				
	168	74.7				

*Note*: BIC, Bayesian Information Criterion; LMR-LRT, Lo-Mendell-Rubin likelihood ratio test. Lower BIC values and higher entropy values (≥0.80) indicate better model fit and classification quality. The 3-class model was selected as optimal based on the following criteria: (1) the lowest BIC value (5668.97); (2) high entropy (0.91), indicating excellent class separation; and (3) a significant LMR-LRT comparing the 3-class to the 2-class model (*p* < 0.001). Models with four classes were also tested but did not converge properly and resulted in one empty class (*n* = 0); therefore, they were not considered further.

**Table 2:** Mean (SD) Barthel Index scores across trajectory classes at four time points.

		Mean (SD)			
Class	*n*	T0	T1	T2	T3
1	46	1.07 (1.77)	3.80 (3.09)	13.22 (4.81)	15.37 (3.22)
2	11	4.09 (7.12)	4.18 (5.79)	3.45 (4.8)	2.36 (1.96)
3	168	10.04 (5.2)	17.7 (3.41)	19.41 (1.41)	19.53 (1.52)

**Table 3:** Associations between recovery trajectories and sociodemographic and clinical factors.

Characteristics	Overall *n* = 225	Class 1 *n* = 46	Class 2 *n* = 11	Class 3 *n* = 168	*p* Value
Sociodemographic factors					
Age, mean (SD)	66.79 (12.53)	69.57 (12.52) ^ab^	78.27 (8.46) ^a^	65.28 (12.28) ^b^	0.001
Gender, male (*n*, %)	134 (59.6)	27 (58.7) ^a^	2 (18.2) ^b^	105 (62.5) ^a^	0.015
Relationship, married/partnered (*n*, %)	170 (75.6)	34 (73.9)	5 (45.4)	131 (78.0)	0.050
Place of residence, town (*n*, %)	154 (68.4)	27 (58.7)	7 (63.6)	120 (71.4)	0.242
Clinical factors					
Comorbidities, yes (*n*, %)	207 (92.0)	44 (95.6)	11 (100.0)	152 (90.5)	0.313
Thrombolysis, yes (*n*, %)	114 (50.7)	25 (54.3)	7 (63.6)	82 (48.8)	0.543
OCSP classification (*n*, %)					<0.001
TACI	35 (15.5)	13 (28.3)	8 (72.7)	14 (8.3)	<0.001
LACI	69 (30.7)	4 (8.7)	2 (18.2)	63 (37.5)	0.006
PACI	90 (40.0)	21 (45.6)	1 (9.1)	68 (40.5)	0.223
POCI	31 (13.8)	8 (17.4)	0 (0.0)	23 (13.7)	0.377
NIHSS at T0, mean (SD)	7.56 (5.21)	11.91 (5.63) ^a^	14.09 (7.44) ^a^	5.94 (3.73) ^b^	<0.001

*Note*: Groups sharing the same superscript letter do not differ significantly in post hoc comparisons, whereas groups with different superscript letters differ significantly (*p* < 0.05). Trajectory classes: Class 1—moderate upward trajectory; Class 2—low-stable trajectory; Class 3—high-functioning stable trajectory. *Abbreviations*: OSCP, The Oxfordshire Community Stroke Project; TACI: total anterior circulation infarct; LACI: lacunar infarct; PACI: partial anterior circulation infarct; POCI, posterior circulation infarct NIHSS, National Institutes of Health Stroke Scale.

**Table 4:** Associations between recovery trajectories and patients’ quality of life and caregiver burden at 3- and 12-month follow-ups.

Characteristics	Overall	Class 1	Class 2	Class 3	*p* Value
	Mean (SD)	Mean (SD)	Mean (SD)	Mean (SD)	
Patients’ SS-QOL	*n* = 225	*n* = 46	*n* = 11	*n* = 168	
Physical dimension at T2	4.11 (0.86)	3.15 (0.74) ^a^	2.19 (0.72) ^b^	4.46 (0.51) ^c^	<0.001
Psychosocial dimension at T2	3.75 (0.82)	3.18 (0.62) ^a^	2.60 (0.69) ^a^	3.96 (0.76) ^b^	<0.001
Total score at T2	3.96 (0.80)	3.15 (0.64) ^a^	2.35 (0.52) ^b^	4.25 (0.57) ^c^	<0.001
Physical dimension at T3	4.22 (0.84)	3.38 (0.65) ^a^	1.86 (0.33) ^b^	4.53 (0.56) ^c^	<0.001
Psychosocial dimension at T3	3.74 (0.91)	3.04 (0.63) ^a^	2.38 (0.58) ^a^	3.98 (0.84) ^b^	<0.001
Total score at T3	4.02 (0.82)	3.23 (0.58) ^a^	2.08 (0.33) ^a^	4.31 (0.63) ^b^	<0.001
Caregiver burden	*n* = 126	*n* = 39	*n* = 11	*n* = 76	
CBS at T2	1.82 (0.72)	2.30 (0.72) ^a^	2.33 (0.75) ^a^	1.50 (0.52) ^b^	<0.001
	*n* = 118	*n* = 37	*n* = 10	*n* = 71	
CBS at T3	1.95 (0.74)	2.39 (0.68) ^a^	2.67 (0.66) ^a^	1.62 (0.59) ^b^	<0.001

*Note*: Groups sharing the same superscript letter do not differ significantly in post hoc comparisons, whereas groups with different superscript letters differ significantly (*p* < 0.05). *Abbreviations*: SS-QOL, Stroke-Specific Quality of Life; CBS, Caregiver Burden Scale; Trajectory classes: Class 1—moderate upward trajectory; Class 2—low-stable trajectory; Class 3—high-functioning stable trajectory.

## Data Availability

The data presented in this study are available on request from the corresponding author due to respondents’ privacy.

## References

[1] Feigin V.L., Brainin M., Norrving B., Martins S.O., Pandian J., Lindsay P.F., Grupper M., Rautalin I. (2025). World Stroke Organization: Global Stroke Fact Sheet 2025. Int. J. Stroke.

[2] Gil-Salcedo A., Dugravot A., Fayosse A., Jacob L., Bloomberg M., Sabia S., Schnitzler A. (2022). Long-term evolution of functional limitations in stroke survivors compared with stroke-free controls: Findings from 15 years of follow-up across 3 International Surveys of Aging. Stroke.

[3] Jaracz K., Grabowska-Fudala B., Jaracz J., Moczko J., Kleka P., Pawlicka A., Górna K. (2024). Caregiver burden after stroke: A 10-year follow-up study of Polish caregivers for stroke patients. BMC Nurs..

[4] Sun Y.A., Kalpakavadi S., Prior S., Thrift A.G., Waddingham S., Phan H., Gall S.L. (2023). Socioeconomic status and health-related quality of life after stroke: A systematic review and meta-analysis. Health Qual. Life Outcomes.

[5] Li S. (2023). Stroke Recovery is a journey: Prediction and potentials of motor recovery after a stroke from a practical perspective. Life.

[6] Aked J., Delavaran H., Wennerström F., Lindgren A.G. (2024). Recovery, functional status, and health-related quality of life status up to 4 years after first-ever stroke onset: A population-based study. Neuroepidemiology.

[7] Walker M.F., Sunnerhagen K.S., Fisher R.J. (2013). Evidence-based community stroke rehabilitation. Stroke.

[8] Gittler M., Davis A.M. (2018). Guidelines for adult stroke rehabilitation and recovery. JAMA.

[9] Levin M.F., Kleim J.A., Wolf S.L. (2009). What do motor “recovery” and “compensationg” mean in patients following stroke?. Neurorehabilit. Neural Repair.

[10] Buvarp D., Viktorisson A., Axelsson F., Lehto E., Lindgren L., Lundström E., Sunnerhagen K.S. (2023). Physical activity trajectories and functional recovery after acute stroke among adults in Sweden. JAMA Netw. Open.

[11] Veerbeek J.M., Kwakkel G., Van Wegen E.E.H., Ket J.C.F., Heymans M.W. (2011). Early prediction of outcome of activities of daily living after stroke: A systematic review. Stroke.

[12] Ganesh A., Luengo-Fernandez R., Rothwell P.M. (2020). Late functional improvement and 5-year poststroke outcomes: A population-based cohort study. J. Neurol. Neurosurg. Psychiatry.

[13] Grefkes C., Fink G.R. (2020). Recovery from stroke: Current concepts and future perspectives. Neurol. Res. Pract..

[14] Woo S.E., Hofmans J., Wille B., Tay L. (2024). Person-centered modeling: Techniques for studying associations between people rather than variables. Annu. Rev. Organ. Psychol. Organ. Behav..

[15] García-Rudolph A., Saurí J., Cegarra B., Madai V.I., Frey D., Kelleher J.D., Cisek K., Opisso E., Tormos J.M., Bernabeu M. (2022). Long-term trajectories of motor functional independence after ischemic stroke in young adults: Identification and characterization using inpatient baseline assessments. NeuroRehabilitation.

[16] Liu L., Li X., Marshall I.J., Bhalla A., Wang Y., O’Connell M.D.L. (2023). Trajectories of depressive symptoms 10 years after stroke and associated risk factors: A prospective cohort study. Lancet.

[17] Hu M., Zhang B., Lin Y., Xu M., Zhu C. (2023). Trajectories of post-stroke quality of life and long-term prognosis: Results from an eleven-year prospective study. J. Psychosom. Res..

[18] Pucciarelli G., Lee C.S., Lyons K.S., Simeone S., Alvaro R., Vellone E. (2019). Quality of life trajectories among stroke survivors and the related changes in caregiver outcomes: A growth mixture study. Arch. Phys. Med. Rehabil..

[19] Huang H.C., Chang C.H., Lee T.H., Chang S.J., Chang T.Y., Huang K.L., Liu C.H., Chang H.J. (2013). Differential trajectory of functional recovery and determinants for first time stroke survivors by using a LCGA approach: A hospital based analysis over a 1-year period. Eur. J. Phys. Rehabil. Med..

[20] Du J., Zhai Y., Dong W., Che B., Miao M., Peng Y., Ju Z., Xu T., He J., Zhang Y. (2024). One-Year Disability Trajectories and long-term cardiovascular events, recurrent stroke, and mortality after ischemic stroke. J. Am. Heart Assoc..

[21] Keeney T., Kumar A., Erler K.S., Karmarkar A.M. (2022). Making the case for patient-reported outcome measures in big-data rehabilitation research: Implications for optimizing patient-centered care. Arch. Phys. Med. Rehabil..

[22] Norrving B., Barrick J., Davalos A., Dichgans M., Cordonnier C., Guekht A., Kutluk K., Mikulik R., Wardlaw J., Richard E. (2018). Action Plan for Stroke in Europe 2018–2030. Eur. Stroke J..

[23] Adams H.P., Davis P.H., Leira E.C., Chang K.-C., Bendixen B.H., Clarke W.R., Woolson R.F., Hansen M.D. (1999). Baseline NIH Stroke Scale score strongly predicts outcome after stroke: A report of the Trial of Org 10172 in Acute Stroke Treatment (TOAST). Neurology.

[24] Brott T., Adams H.P., Olinger C.P., Marle J.R., Barsan W.G., Biller J., Spilker J., Holleran R., Eberle R., Hertzberg V. (1989). Measurements of acute cerebral infarction: A clinical examination scale. Stroke.

[25] Wade D.T., Hewer R.L., David R.M., Enderby P. (1986). Aphasia after stroke: Natural history and associated deficits. J. Neurol. Neurosurg. Psychiatry.

[26] Collin C., Wade D.T., Davies S., Horne V. (1988). The Barthel ADL index: A reliability study. Int. Disabil. Stud..

[27] Williams L.S., Weinberger M., Harris L.E., Clark D.O., Biller J. (1999). Development of a stroke-specific quality of life scale. Stroke.

[28] Bejer A., Kwolek A. (2009). Polish adaptation of stroke-specific quality of life scale. Adv. Rehabil..

[29] Elmståhl S., Malmberg B., Annerstedt L. (1996). Caregiver’s burden of patients 3 years after stroke assessed by a novel caregiver burden scale. Arch. Phys. Med. Rehabil..

[30] Jaracz K., Grabowska-Fudala B., Kleka P., Smelkowska A., Pawlicka A., Górna K., Tomczak M. (2022). Development and psychometric properties of the Caregiver Burden Scale in Polish caregivers of stroke patients. Psychol. Res. Behav. Manag..

[31] Jung T., Wickrama K.A.S. (2008). An Introduction to Latent Class Growth Analysis and Growth Mixture Modeling. Soc. Pers. Psychol. Compass.

[32] Tein J.-Y., Coxe S., Cham H. (2013). Statistical power to detect the correct number of classes in latent profile analysis. Struct. Equ. Model. SEM.

[33] TIBCO Software Inc (2017). TIBCO Statistica (Data Analysis Software System).

[34] R Core Team (2025). R: A Language and Environment for Statistical Computing.

[35] Ganesh A., Luengo-Fernandez R., Wharton R.M., Gutnikov S.A., Silver L.E., Mehta Z., Rothwell P.M. (2017). Time course of evolution of disability and cause-specific mortality after ischemic stroke: Implications for trial design. J. Am. Heart Assoc..

[36] Wurzinger H.E., Abzhandadze T., Rafsten L., Sunnerhagen K.S. (2021). Dependency in Activities of Daily Living During the First Year After Stroke. Front. Neurol..

[37] Guo X., Xiong Y., Huang X., Pan Z., Kang X., Chen C., Zhou J., Zheng H., Chen Y., Hu W. (2023). Sex-based differences in long-term outcomes after stroke: A meta-analysis. PLoS ONE.

[38] Gargano J.W., Reeves M.J. (2007). Sex differences in stroke recovery and stroke-specific quality of life: Results from a statewide stroke registry. Stroke.

[39] Yun S.M., Shin S., Chang W.H., Kim D.Y., Lee J., Sohn M.K., Song M.K., Shin Y.I., Lee Y.S., Joo M.C. (2023). Gender differences in mortality and long-term functional outcomes after first-ever ischemic stroke: A prospective cohort study. Int. J. Stroke.

[40] Ali M., van Os H.J.A., van der Weerd N., Schoones J.W., Heymans M.W., Kruyt N.D., Visser M.C., Wermer M.J.H. (2022). Sex differences in presentation of stroke: A systematic review and meta-analysis. Stroke.

[41] Irie F., Nakamura K., Matsuo R., Wakisaka Y., Ago T., Kitazono T., Kamouchi M. (2025). Fukuoka Stroke Registry Investigators. Factors related to sex differences in long-term functional decline after acute ischemic stroke. Sci. Rep..

[42] Owais S.B., Bulwa Z.B., Ammar F. (2024). El Differences in stroke clinical presentation among sexes. J. Stroke Cerebrovasc. Dis..

[43] Xu M., Amarilla Vallejo A., Cantalapiedra Calvete C., Rudd A., Wolfe C., O’connell M.D.L., Douiri A. (2022). Stroke outcomes in women: A population-based cohort study. Stroke.

[44] Yoo J.W., Hong B.Y., Jo L., Kim J.S., Park J.G., Shin B.K., Lim S.H. (2020). Effects of age on long-term functional recovery in patients with stroke. Medicina.

[45] Knoflach M., Matosevic B., Rücker M., Furtner M., Mair A., Wille G., Zangerle A., Werner P., Ferrari J., Schmidauer C. (2012). Functional recovery after ischemic stroke--a matter of age: Data from the Austrian Stroke Unit Registry. Neurology.

[46] Ohya Y., Matsuo R., Sato N., Irie F., Wakisaka Y., Ago T., Kamouchi M., Kitazono T. (2023). Modification of the effects of age on clinical outcomes through management of lifestyle-related factors in patients with acute ischemic stroke. J. Neurol. Sci..

[47] Zhu C., Tran P.M., Leifheit E.C., Spatz E.S., Dreyer R.P., Nyhan K., Wang S.Y., Roberson P.N.E., Goldstein L.B., Lichtman J.H. (2025). Association between marital/partner status and patient-reported outcomes in stroke patients: A systematic review. Cerebrovasc. Dis..

[48] Liu Q., Wang X., Wang Y., Wang C., Zhao X., Liu L., Li Z., Meng X., Guo L., Wang Y. (2018). Association between marriage and outcomes in patients with acute ischemic stroke. J. Neurol..

[49] Dedijer Dujović S., Djordjević O., Vidaković A., Mitrović S., Grajić M., Tomić T.D., Rosić S., Radić A., Konstantinović L. (2025). Inequities in stroke recovery: Examining sociodemographic predictors of rehabilitation success. Healthcare.

[50] Anderson S., Keating N. (2018). Marriage after the transition to stroke: A systematic review. Ageing Soc..

[51] Du J., Wang Y., Che B., Miao M., Bao A., Peng Y., Ju Z., Xu T., He J., Zhang Y. (2023). The relationship between neurological function trajectory, assessed by repeated NIHSS measurement, and long-term cardiovascular events, recurrent stroke, and mortality after ischemic stroke. Int. J. Stroke.

[52] Rost N.S., Bottle A., Lee J.M., Randall M., Middleton S., Shaw L., Thijs V., Rinkel G.J.E., Hemmen T.M., Global Comparators Stroke GOAL Collaborators (2016). Stroke severity is a crucial predictor of outcome: An international prospective validation study. J. Am. Heart Assoc..

[53] Paci M., Nannetti L., D’Ippolito P., Lombardi B. (2011). Outcomes from ischemic stroke subtypes classified by the Oxfordshire Community Stroke Project: A systematic review. Eur. J. Phys. Rehabil. Med..

[54] Reggiani M., Società Inter-Regionale Piemonte e Valle d’Aosta per le Cerebrovasculopatie Group (2009). Five-year survival after first-ever ischaemic stroke is worse in total anterior circulation infarcts: The SINPAC cohort. Cerebrovasc. Dis..

[55] Miao Y., Li X., Wang S., Li C. (2026). The value and limitations of the Oxford Community Stroke Project classification in the hyperacute phase of cerebral infarction. Front. Neurol..

[56] Petty G.W., Brown R.D., Whisnant J.P., Sicks J.D., O’Fallon W.M., Wiebers D.O. (2000). Ischemic stroke subtypes: A population-based study of functional outcome, survival, and recurrence. Stroke.

[57] Garcia D.J., Makin S.D.J., McHutchison C.A., Cvoro V., Valdés Hernández M.D.C., Sakka E., Chappell F.M., Doubal F., Wardlaw J.M. (2025). Functional, cognitive, physical, and vascular outcomes 9 years after lacunar and mild cortical ischemic stroke. Neurology.

[58] Yu C., An Z., Zhao W., Wang W., Gao C., Liu S., Wang J., Wu J. (2015). Sex Differences in Stroke Subtypes, Severity, Risk Factors, and Outcomes among Elderly Patients with Acute Ischemic Stroke. Front. Aging Neurosci..

[59] Nacu A., Fromm A., Sand K.M., Waje-Andreassen U., Thomassen L., Naess H. (2016). Age dependency of ischaemic stroke subtypes and vascular risk factors in western Norway: The Bergen Norwegian Stroke Cooperation Study. Acta Neurol. Scand..

[60] van Nimwegen D.J.J., Vervoort S.C.J.M., Visser-Meily J.M.A., Schoonhoven L., de Man-van Ginkel J.M. (2025). Reshaping life after stroke: A grounded theory. Int. J. Nurs. Stud. Adv..

[61] Liu Z., Xiang D., Ge S., Mei Y., Zhang Z., Chen S., Guo E., Li X. (2025). Trajectories of dyadic quality of life in young to middle-aged stroke couples: A longitudinal study. Qual. Life Res..

[62] Bartoli D., Brugnera A., Grego A., Alvaro R., Vellone E., Pucciarelli G. (2024). Stroke disease–specific quality of life trajectories and their associations with caregivers’ anxiety, depression, and burden in stroke population: A longitudinal, multicentre study. Eur. J. Cardiovasc. Nurs..

[63] van Mierlo M., van Heugten C., Post M.W.M., Hoekstra T., Visser-Meily A. (2018). Trajectories of health-related quality of life after stroke: Results from a one-year prospective cohort study. Disabil. Rehabil..

[64] Pucciarelli G., Ausili D., Galbussera A.A., Rebora P., Savini S., Simeone S., Alvaro R., Vellone E. (2018). Quality of life, anxiety, depression and burden among stroke caregivers: A longitudinal, observational multicentre study. J. Adv. Nurs..

